# Modeling and Enhancing Responsivity for the InGaAsN/GaAs Configuration in p-i-n Photodiodes

**DOI:** 10.3390/mi17101160

**Published:** 2026-09-30

**Authors:** Nabil Hafi, Abdelkader Aissat, Kacim Hadjadj, Samuel Dupont, Faouzi Saidi, Mohamed Houcine Dhaou, Jean Pierre Vilcot

**Affiliations:** 1 LATSI Laboratory, University Blida1, Blida 09000, Algeria; nabil_elec@yahoo.fr; 2 Department of Material Science, Faculty of Material Science, Mathematics and Computer Science, University of Ahmed Draya, Adrar 01000, Algeria; kacim.hadjadj@univ-adrar.edu.dz; 3 Institute of Electronics, Microelectronics and Nanotechnology (IEMN), UMR CNRS 8520, University of Sciences and Technologies of Lille, 1 Avenue Poincare, 60069, 59652 Villeneuve of Ascq, France; samuel.dupont@uphf.fr (S.D.); jean-pierre.vilcot@univ-lille.fr (J.P.V.); 4 Micro-Optoelectronics and Nanostructures Laboratory, Department of Physics, Faculty of Sciences of Monastir, University of Monastir, Environment Street, Monastir 5000, Tunisia; faouzi.saidi@gmail.com; 5 Department of Physics, College of Science, Qassim University, P.O. Box 51452, Burayda Almolaydah 6644, Saudi Arabia; m.dhaou@edu.sa

**Keywords:** materials, semiconductor, III-V-N, stable structure, responsivity, detection, optoelectonic

## Abstract

This paper presents the numerical modeling, simulation, and optimization of a p-i-n photodiode (PD) based on quaternary InGaAsN using the Atlas Silvaco simulator. The proposed architecture incorporates wide-bandgap, transparent GaAs contact layers on both the p and n sides to enhance optical coupling and frequency response characteristics. For the unstrained configuration (In = 6%, N = 2%, ε = 0%, and T = 300 K) with an absorber thickness of d_abs_ = 1.5 µm under a −2 V reverse bias, the device achieves a peak responsivity R(λ) of 0.339 A/W at a wavelength of 0.975 µm, corresponding to a quantum efficiency (QE) of 43% and a cutoff frequency (F_c_) of 11.60 GHz. To evaluate the influence of mechanical strain, a comparative analysis was performed for a strained structure (In = 10%, N = 2%, ε = −0.30%), which exhibited a degraded QE of 38.47%. The simulated results for the unstrained photodiode show strong agreement with benchmark data from the literature. This comparison demonstrates that the proposed strain-free p-i-n PD structure achieves improved optoelectronic performance, supporting its potential for high-speed photodetection applications.

## 1. Introduction

III-V nitride-based semiconductors (SC) have significant potential in photonic and optical components relevant to power electronics and high-frequency devices, including LEDs [1,2] and transistors designed for high power and frequency [3], photodiodes (PDs) and solar cells [4,5]. Heterostructures, particularly those utilizing III-V quaternary compounds, have capitalized on these developments and have become important candidates for optoelectronic devices due to their appealing physical and optical characteristics [6].

Since the initial proposal by Kondow et al. [7], InGaNAs has garnered significant attention due to its suitability for use in optoelectronic devices such as lasers and detectors operating within the 1.3 to 1.7 µm wavelength range. InGaNAs stands out from other semiconductor alloys because the incorporation of small amounts of nitrogen (N) significantly alters its physical properties by narrowing the bandgap and increasing its responsivity to infrared light [8]. Among the alloys studied in recent years, InGaAsN deposited on GaAs has received particular attention because it allows for narrow bandgap widths on GaAs substrates [9]. Furthermore, InGaAsN can match the crystal lattice of GaAs with a concentration ratio [In]/[N] of around three. This condition helps overcome the existing thickness limitation in strain-compensated epitaxy of GaInAs layers on GaAs substrates. This new alloy family brings significant advancement to heterostructure engineering fabricated on GaAs and InGaAs [10,11,12].

To enhance the responsivity R(λ) of p-i-n photodiodes (PDs), extensive research has focused on InGaN/GaN and InGaAsN/GaAs heterostructures for high-speed photodetection. For instance, Berkman et al. [13] achieved an R(λ) of 0.037 A/W at 0.426 µm under zero-bias conditions of the InGaN p-i-n PD. Notably, Su et al. [14] reported a peak R(λ) of 0.206 A/W at λ = 0.380 µm for an In_0.11_Ga_0.89_N p-i-n PD, while Lu et al. [15] obtained a maximum R(λ) of 0.220 A/W at λ = 0.378 µm in an unbiased InGaN p-i-n PD. More recently, Huang et al. [16] designed a UV p-i-n PD made of In_0.09_Ga_0.91_N and performed simulations with the Atlas-Silvaco tool; this resulted in a peak R(λ) of 0.190 A/W at λ = 0.402 µm. To achieve high-speed performance, an InGaN/GaN MQW p-i-n PD with a cutoff frequency (F_c_) of 390 MHz [17] has been achieved. A p-i-n PD was created [18] with a 0.100 µm thick layer of In_0.1_Ga_0.9_N as the absorption region, achieving a F_c_ of 400 MHz. However, increasing the thickness of the intrinsic region (d_abs_) to 1.5 µm resulted in a F_c_ of 4 GHz. Xie et al. [19] reported a peak R(λ) of 0.180 A/W at λ = 395 nm and under −10 V bias, corresponding to a maximum QE of 56%. Suram and Gupta [20] designed and optimized a photodetector with a QE exceeding 50% and demonstrated high responsivity, indicating its potential for efficient operation in the UV wavelength range. Saidani and Tobbeche [21] demonstrated that the GaN-based p-i-n heterostructure outperformed its homojunction counterpart, achieving a peak R(λ) of 0.300 A/W at λ = 0.363 μm under specific conditions.

In this context, recent studies have demonstrated significant improvements in photodetector performance through material engineering, defect suppression, and device architecture optimization. Wu et al. [22] developed bismuth–antimony halide-based PD with enhanced responsivity, while Song et al. [23,24] achieved high-performance CsCu_2_I_3_/GaN UV photodetectors via epitaxial growth and thermal sublimation. These advances underscore the critical importance of tailoring material properties and device architectures, thereby motivating the investigation of alternative systems such as the InGaAsN/GaAs p-i-n photodiode, which shows particular promise for near-infrared photodetection. In previous studies (Ref. [6]), the investigation was strictly targeted at optimizing the frequency response and high-speed operation of a lattice-matched InGaAsN/GaAs p-i-n photodiode for 1.15 µm detection, specifically through incorporating dual transparent layers and adjusting the depletion region thickness to balance cutoff frequency, junction capacitance, and quantum efficiency. Similarly, in earlier work on InGaAs/InP photodiodes [25], the focus was primarily placed on analyzing and optimizing the frequency response and cutoff frequency of a p-i-n structure as a function of strain, indium content, and device geometry. Specifically, the present design utilizes the InGaAsN/GaAs p-i-n architecture reported in previous work [6], employing an intrinsic InGaAsN active layer lattice-matched to the GaAs substrate. The design further incorporates two transparent wide-bandgap GaAs cladding layers positioned on the p and n sides of the device to minimize parasitic absorption, with numerical investigations carried out using the Atlas Silvaco simulator. In the first phase of our study, we investigated the effect of the InGaAsN absorbing layer thickness on the spectral responsivity R(λ) of the lattice-matched photodiode (In = 6%, N = 2%, ε = 0%). We then examined the spectral response by varying the thickness of the p-GaN layer (w_p_) while keeping the absorber thickness fixed at d_abs_ = 1.5 µm and the n-GaN layer at w_n_ = 1 µm. A similar parametric evaluation was conducted by varying w_n_ with d_abs_ = 1.5 µm and w_p_ = 1 µm. In the second phase, we evaluated the influence of epitaxial strain by comparing the responsivity of the unstrained structure against the compressive-strained configuration. Finally, we analyzed the frequency response (FR) of the stable p-i-n PD structure, i.e., without strain, at room temperature (T = 300 K) with d_abs_ = 1.5 µm.

## 2. Materials and Methods

The structure used in this study is based on the In_u_Ga_1−u_As_1−v_N_v_/GaAs system, where u and v represent the indium (In) and nitrogen (N) concentrations, respectively. This material composition (u,v) is of particular interest due to its ability to be grown in lattice matching with the GaAs substrate. This lattice matching enables the maintenance of a stable structure ε(u,v) = 0, which means that the strains induced by differences in lattice parameters are minimized, where ε(u,v) = 0 signifies the strain given by [26,27]. The band anti-crossing (BAC) model may be utilized to calculate the unstrained bandgap energy [6,26,27,28]:

(1) $$E\pm \left(v\right)=0.5\left[\left(E_{N}(v)+E_{M}\right)\pm \sqrt{{\left(E_{N}(v)+E_{M}\right)}^{2}+4V_{MN}^{2}(v)}\right]$$

(2) $$E_{N}(v)=1.52-3.9v$$

(3) $$E_{M}=E_{0}-1.55eV+\frac{h^{2}}{2m_{e}^{*}}$$

(4) $$V_{MN}(v)=C_{MN}\sqrt{v}$$

In this formulation, C_MN_ = 2.3 eV is the hybridization coupling parameter. The term E_0_ denotes the conduction band (CB) minimum of the host InGaAs ternary alloy in the absence of nitrogen, while $m_{e}^{*}$ represents the electron effective mass in InGaAs. Furthermore, E_M_ corresponds to the CB edge energy of the InGaAs matrix, and E_N_ is the localized nitrogen level referenced to the valence band (VB) maximum. Finally, V_MN_ represents the interaction matrix element that describes the hybridization coupling between the host matrix states E_M_ and the localized nitrogen states E_N_.

Examine how a spatially localized nitrogen level (denoted E_N_) interacts with the continuous states of the CB of the host material in a two-band E_+_ and E_-_; this interaction is explained by the BAC model’s Hamiltonian [29]. The calculated bandgap energy (E_g_) of the strained material is given by the following equation [26,30]:

(5) $$E_{g}^{strain}(u,v)=E_{g}^{unstrain}(u,v)+{∆E}_{c}^{hy}(u,v)+{∆E}_{v,av}^{hy}(u,v)-{∆E}_{hh}^{sh}(u,v)$$

where $E_{g}^{strain}(u,v)$ denotes the bandgap under deformation conditions, whereas $E_{g}^{unstrain}(u,v)$ refers to the band gap in the absence of deformation.

${∆E}_{c}^{hy}(u,v)$, ${∆E}_{v,av}^{hy}(u,v)$ are the gravitational centers’ energy offsets in the VB and CB, respectively.

(6) $${∆E}_{c}^{hy}(u,v)=2a_{c}(u,v)\left(1-\frac{C_{12}(u,v)}{C_{11}(u,v)}\right)\varepsilon (u,v)$$

(7) $${∆E}_{v,av}^{hy}(u,v)=-2a_{v}(u,v)\left(1-\frac{C_{12}(u,v)}{C_{11}(u,v)}\right)\varepsilon (u,v)$$

${∆E}_{hh}^{sh}(u,v)$ is the shear strain-induced energy offset in the heavier holes [23].

(8) $${∆E}_{hh}^{sh}(u,v)=-b(u,v)\left(1+\frac{2C_{12}\left(u,v\right)}{C_{11}(u,v)}\right)\varepsilon (u,v)$$

The parameters a_c_(u,v) and a_v_(u,v) correspond to the VB and CB respective potentials for hydrostatic deformation, while b(u,v) denotes the valence band’s shear deformation potential, and C_ij_(u,v) represents the elastic Rigidity constants, which depend on the concentration (u,v). To determine these factors for our quaternary material, we implemented Vegard’s law. Table 1 contains the parameter values used throughout this research study [26,31]. While an SC layer is growing, when the lattice parameter a_e_(u,v) differs from that of the substrate a_s_, we observe two types of strains: compressive strain if (a_e_(u,v) > a_s_) or tensile strain if (a_e_(u,v) < a_s_)*;* these strains induce a tetragonal deformation of the crystalline lattice [32]. When In_u_Ga_1−u_As_1−v_N_v_ is grown by epitaxy on a substrate (GaAs) with different lattice parameters, it induces deformation in which the absorbent layer is subject to the substrate’s own lattice parameter. This results in strain that is perpendicular to the growth plane and parallel to the absorbent layer plane [33]. The corresponding relationships are formulated using Equations (9) and (11) as follows:

In the plane of the layer, the net strain is given by

(9) $$\varepsilon_{xx}(u,v)=\varepsilon_{yy}(u,v)=\frac{a_{s}-a_{e}(u,v)}{a_{e}(u,v)}$$

where the lattice constants a_s_ and a_e_(u,v) denote the crystalline parameters of GaAs and In_u_Ga_1−u_As_1−v_N_v_, respectively.

In the direction of growth,

(10) $$\varepsilon_{zz}(u,v)=-2\frac{C_{12}(u,v)}{C_{11}(u,v)}\varepsilon_{xx}(u,v)$$

The condition that allows the achievement of a stable structure for each pair (u,v) of In and N concentration is determined by [6].

(11) $$v(u)=\frac{au}{b-cu}$$

where a = 0.40, b = 1.17, and c = 0.075.

The primary function of the PD is to capture photon energy in order to generate an electrical signal. When a semiconductor (SC) is subjected to light radiation with energy (E_ph_), photons that meet the criterion E_ph_ ≥ E_g_ can be absorbed by electrons in the valence band (VB). For E_ph_ exceeding E_g_, the absorption coefficient α(E) is determined by the following expression [6,32,33,34].

(12) $$\alpha (E)=\alpha_{0}\frac{\sqrt{E_{ph}-E_{g}^{strain}(u,v)}}{E_{ph}}$$

where α_0_ is a constant that depends on the SC and $E_{g}^{strain}(u,v)$ is the strain bandgap energy of the In_u_Ga_1−u_As_1−v_N_v_, calculated using Equation (5). For a stable structure with In = 6% and a low nitrogen concentration (N = 2%), the bandgap energy decreases to approximately 1.1 eV [6].

The spectral responsivity R(λ) of a photodiode is typically expressed in amperes per watt (A/W), which indicates the amount of electrical current generated by the PD in response to a certain amount of incident light power, and is given by [35].

(13) $$R(\lambda )=\frac{I_{T}}{I_{s}}\left(\frac{\lambda }{1.24}\right)$$

where I_T_ is the cathode current, I_S_ is the source photocurrent, and λ is the wavelength.

The QE of the photodiode is derived from measured spectral photocurrent data using the following equations [8]:

(14) $$QE(\lambda )=R(\lambda )\left(\frac{hc}{q\lambda }\right)$$

Here, h represents Planck’s constant, c is the speed of light, q is the electron charge, and λ is the wavelength.

The normalized frequency response is defined in decibels (dB) as [10]:

(15) $$R_{dB}(f)=20.log\left(\frac{I_{R}(f)}{I_{R0}}\right)$$

where I_R_(f) is the AC cathode photocurrent, and I_R0_ is its low-frequency value. The cut-off frequency (f_−3dB_) is now formally defined as the frequency where the normalized response drops by −3 dB (corresponding to a current reduction by a factor of $\sqrt{2}\;\approx 0.707$):

(16) $$R_{dB}(f_{-3dB})=20.log\left(\frac{I_{R}(f_{-3dB})}{I_{R0}}\right)=-3\;dB$$

**Table 1:** Parameters of binary materials used in this modeling [26].

Parameter	GaAs	InAs	GaN	InN
a (Å), T = 300 K	5.65	6.06	4.50	4.98
E_g_ (eV), T = 300 K	1.42	0.35	3.40	1.94
∆_0_ (eV)	0.34	0.39	0.02	0.01
a_c_ (eV)	−7.17	−5.08	−2.20	−1.85
a_V_ (eV)	1.16	1.00	−5.20	−1.50
b (eV)	−1.70	−1.80	−2.20	−1.20
C_11_ (GPa)	1221	833	293	187
C_12_ (GPa)	566	453	159	125
α (meV/K)	0.54	0.28	0.909	0.245
β (meV/K)	205	93	830	624
N_c_ (×10^18^ cm^−3^)	0.47	0.086	2.3	0.91
N_v_ (×10^18^ cm^−3^)	0.70	1.00	4.4	5.3
γ_1_	6.80	20.0	2.67	3.72
γ_2_	1.90	8.50	0.75	1.26
γ_3_	2.93	9.20	1.10	1.63

## 3. Results and Discussion

**Figure 1:**
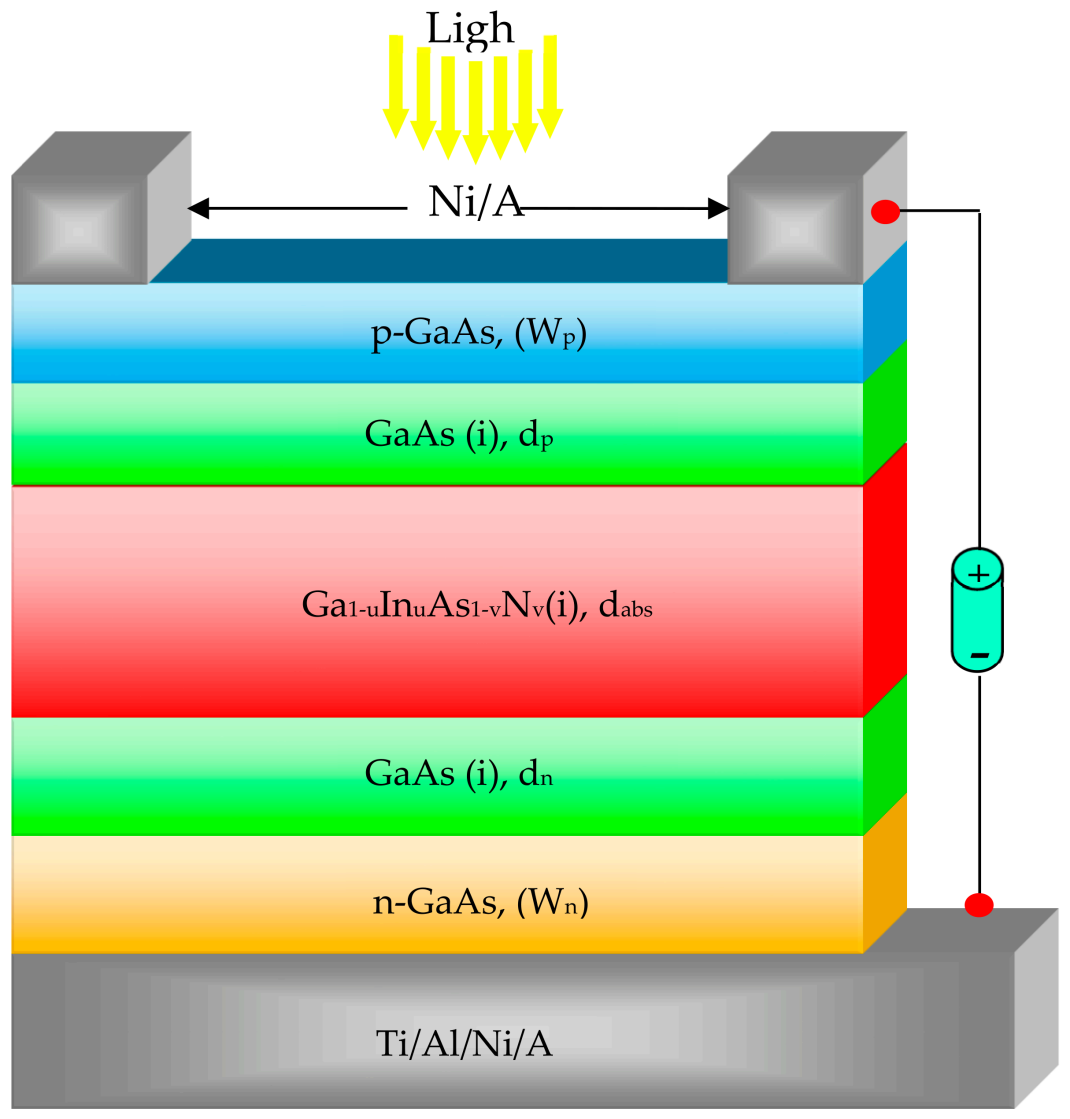
The proposed structure for the InGaAsN/GaAs p-i-n photodiode.

To ensure the performance and lifetime of the structure in use, it is essential to examine the stability of this structure. The proposed InGaAsN/GaAs p-i-n PD structure, based on the architecture reported in previous work [6], incorporates two transparent GaAs regions added on the p and n sides to enhance frequency response characteristics, as shown in Figure 1. The structure comprises an n region with W_n_ = 1 µm and a doping concentration of 1 × 10^18^ cm^−3^, followed by the first underlying field-transparent GaAs region with d_n_= 0.5 µm. The absorbing layer is made of unintentionally doped In_u_Ga_1-u_As_1-v_N_v_ (N_A_ ≈ 10^15^ cm^−3^) with a thickness of d_abs_. Beneath it lies a transparent GaAs region with a thickness of d_p_ = 0.5 µm, followed by a p-region that has a width of w_p_ = 1 µm and a doping concentration of 1 × 10^19^ cm^−3^. The cathode contact consists of Ti/Al/Au, while the anode contact is Ni/Au, covering a surface area of 25 µm^2^ [6]. The incident light hits the photodetector on its p-doped region side.

**Figure 2:**
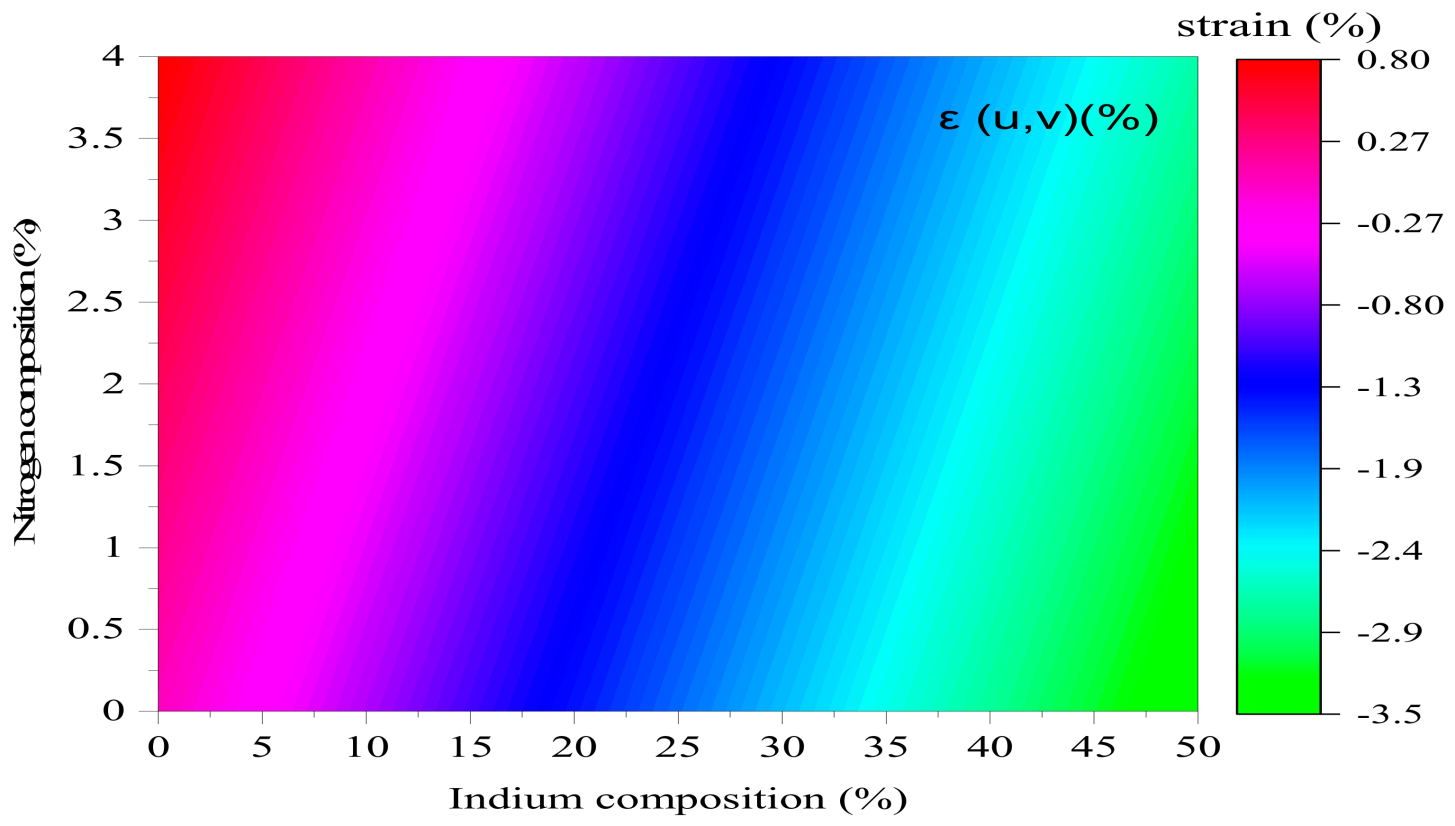
Variation in strain concerning In and N concentrations within the InGaAsN/GaAs p-i-n photodetector structure.

Figure 2 illustrates the variation in strain ε(u,v) with varying concentrations of indium (In) and nitrogen (N) in InGaNAs. It is evident that this material typically experiences compressive strain.

As the concentration of In increases, the strain within the material also escalates. In contrast, a higher concentration of N leads to a reduction in strain. For example, when u = 10% and v = 2%, the compressive strain is measured at 0.30%. If the In concentration is raised to 40% and N to 2%, the strain remains compressive, reaching 2.39%. In an InGaAs alloy, a higher proportion of indium leads to an increase in the lattice parameter relative to the GaAs substrate, causing compressive strain. The addition of nitrogen in InGaAs partially mitigates this compression effect. Once the nitrogen concentration surpasses 3%, the compression reduces, and the structure accommodates it. If the nitrogen concentration is too high, there is a notable rise in defects within the material.

**Figure 3:**
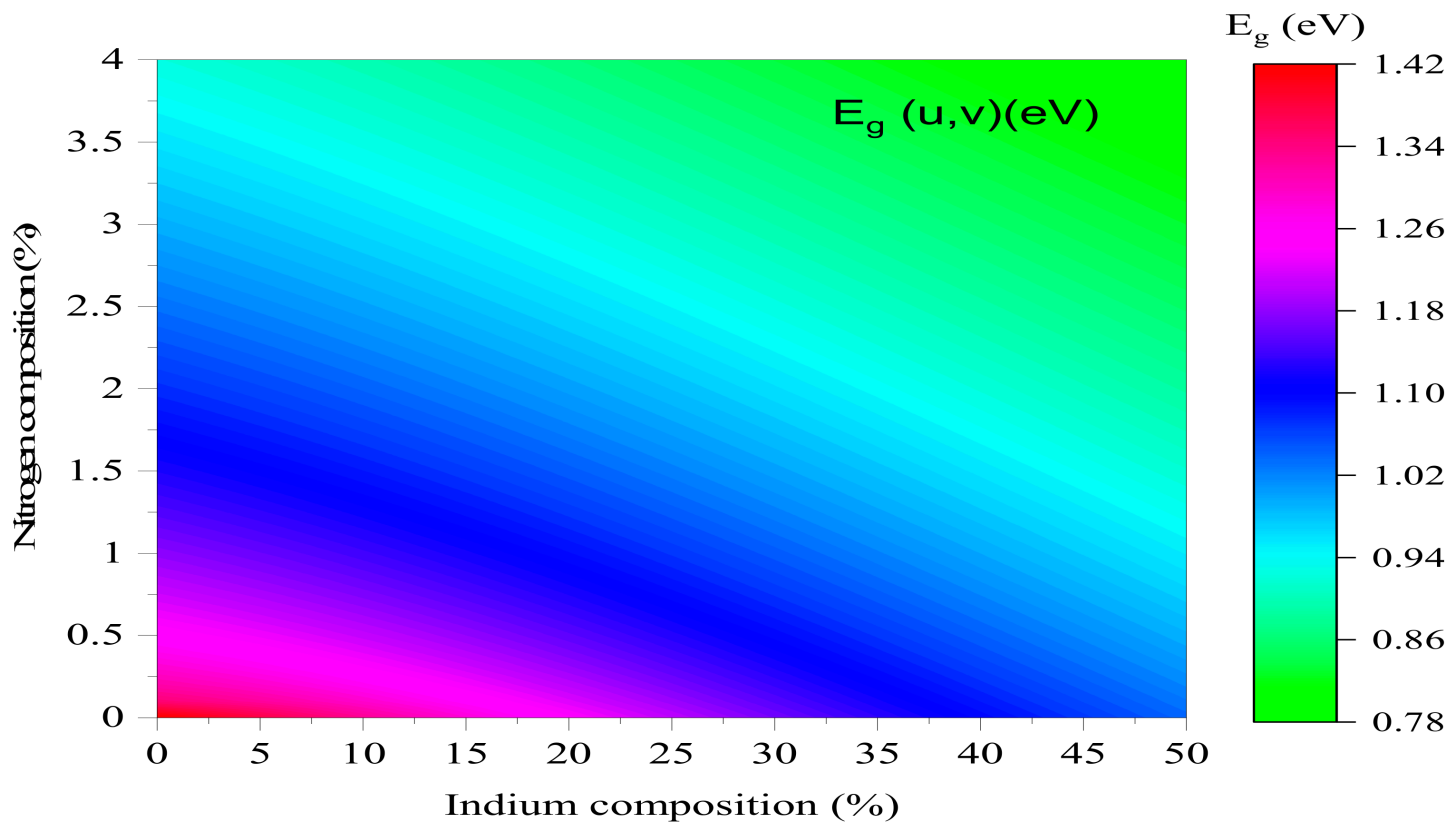
The bandgap energy E_ghh_ as a function of In and N concentrations.

Figure 3 shows the connection between bandgap energy E_ghh_ and different concentrations of In and N in the InGaAsN material. It is clear that increasing concentrations of In and N result in a decrease in E_ghh_. The reduction in E_ghh_ as In content rises is due to alterations in the material’s electronic band structure. Similarly, a higher N concentration introduces additional energy levels within the bandgap, which disrupts the band structure and reduces the bandgap energy. It is observed that, in practice, InGaAsN alloys on GaAs typically contain between 0.5% and 4% nitrogen, as this range effectively reduces the bandgap while maintaining an acceptable crystal quality. Exceeding this limit often leads to growth issues and an increase in defects, which can become limiting factors. The combined influence of these factors enables significant tuning of the bandgap energy, thereby directly impacting the optical and electronic properties of the material [26].

**Figure 4:**
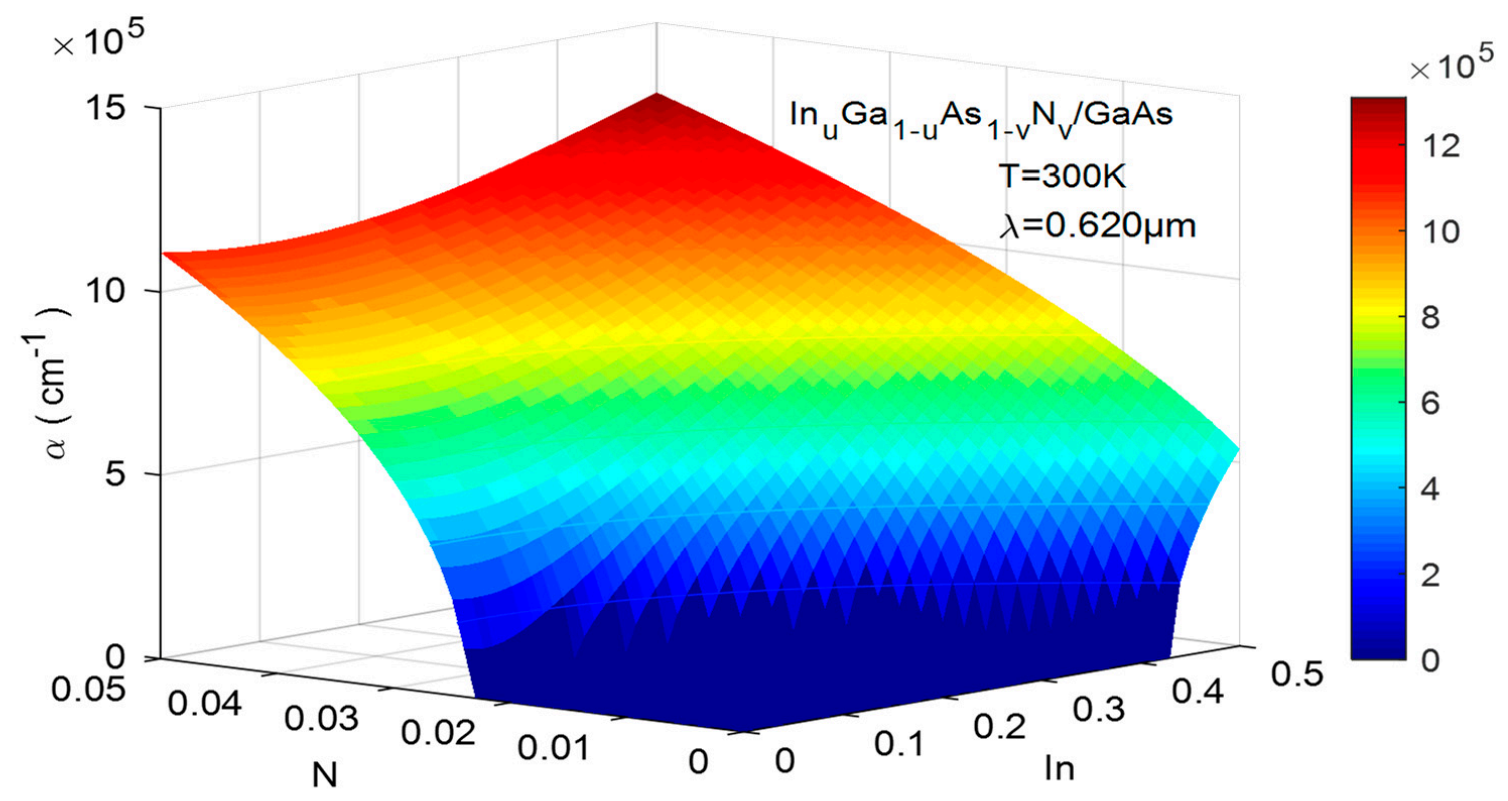
Variation in the absorption coefficient of the InGaAsN structure as a function of concentrations u and v at T = 300 K and λ = 0.620 µm.

The influence of In and N concentrations on the absorption coefficient α at T = 300 K and the incident light wavelength of λ = 0.620 µm was investigated and assessed. For the values (u = 10%, v = 3%), the coefficient *α* reaches 5.70 × 10^5^ cm^−1^ under a compressive strain of ε = 0.27%. When both concentrations are increased to u = 15% and v = 4%, the coefficient α is found to be 8.85 × 10^5^ cm^−1^ with ε = 0.80%. This simulation facilitates the optimization of the absorption coefficient *α*, enabling the selection of the most efficient and stable absorbing layer (Figure 4).

**Figure 5:**
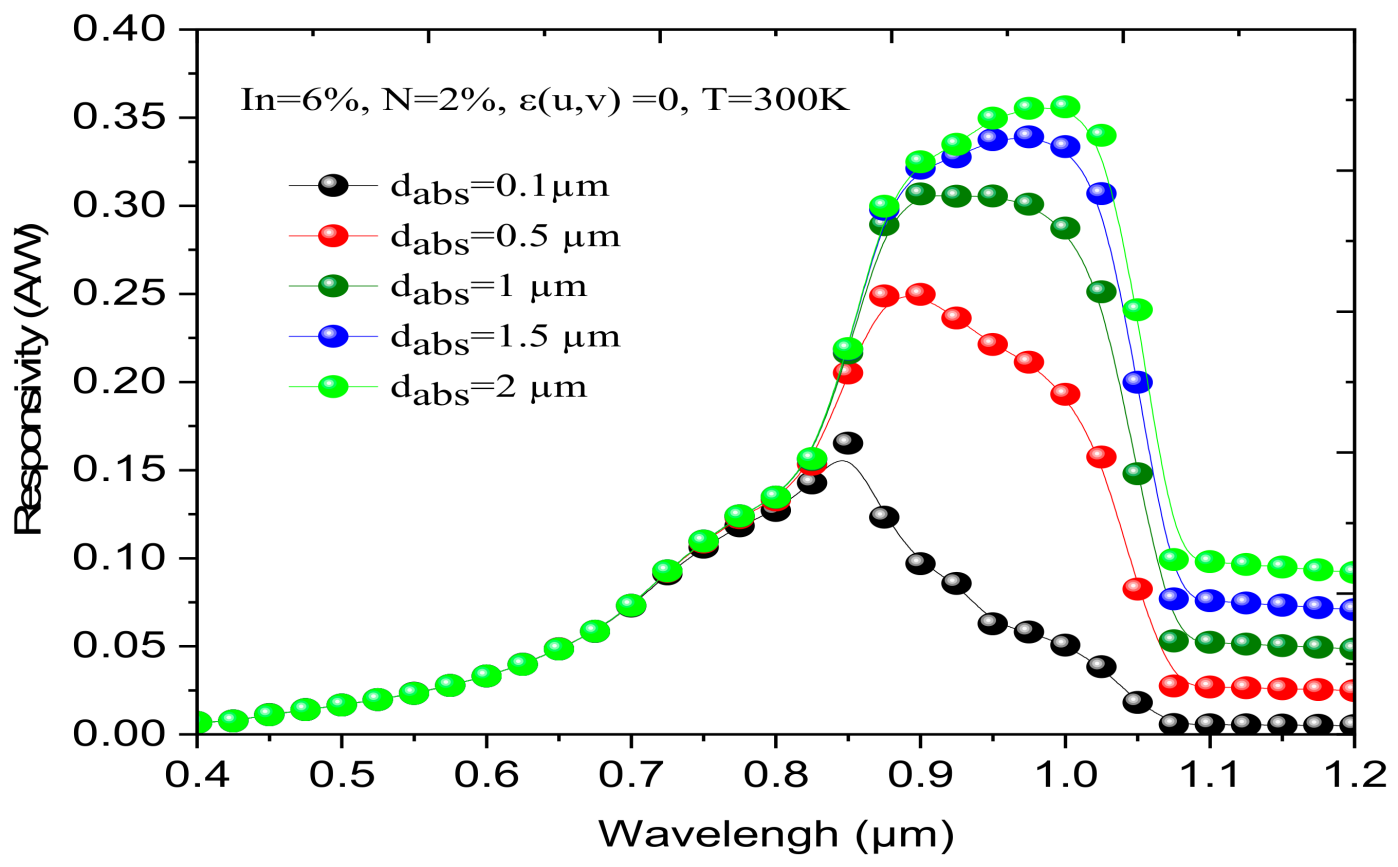
Responsivity of the InGaAsN/GaAs p–i–n photodiode as a function of wavelength for the different absorption layer thicknesses under 2 V reverse bias at room temperature.

Figure 5 illustrates how the sensitivity of the proposed photodiode varies with wavelength for different thicknesses of the absorption layer (d_abs_). This layer consists of 6% In and 2% N with ε(u,v) = 0, under a reverse bias voltage of −2 V at T = 300 K. Within the spectral range of 0.8 to 1 μm, R(λ) increases with dabs, reaching a peak of 0.339 A/W at λ = 0.975 µm. It is important to note that increasing dabs leads to an expansion of the depletion region of the p-i-n photodiode. This increase in responsivity is attributed to a higher probability that the incident photon radiation is absorbed and converted into a measurable signal. We observe that when a photon passes through an absorbing layer, it has a certain probability of interacting with the material. A thicker layer provides a longer path within the material, thereby enhancing this probability. The responsivity of a photodiode refers to its ability to respond to a low radiation flux. Since a thicker layer absorbs a larger proportion of the incident radiation, the photodiode produces a higher signal for the same incident intensity. As the thickness of the absorption layer increases, the depletion region (sensitive zone) expands, allowing a greater number of photons to interact with the material, resulting in the generation of more charge carriers (electron-hole pairs).

Therefore, d_abs_ plays a vital role in the performance and responsiveness of a photodetector (PD). The response speed of the PD is influenced by two primary factors: the transit time of charge carriers (tr) and the RC time constant (which is determined by the total resistance R, load resistance, and capacitance (C)). By increasing dabs, we enhance reactivity through improved light absorption while simultaneously reducing C, a phenomenon that tends to increase bandwidth (BW). However, this increase also leads to a rise in tr, which may limit the enhancement of BW. It is important to note that when the absorption thickness reaches 2 µm, the responsivity begins to stabilize. This saturation phenomenon occurs because charge carriers may recombine before being collected; additionally, thermal noise and collection losses can increase. Therefore, we must find a balance between the absorber thickness and the absorption coefficient.

**Figure 6:**
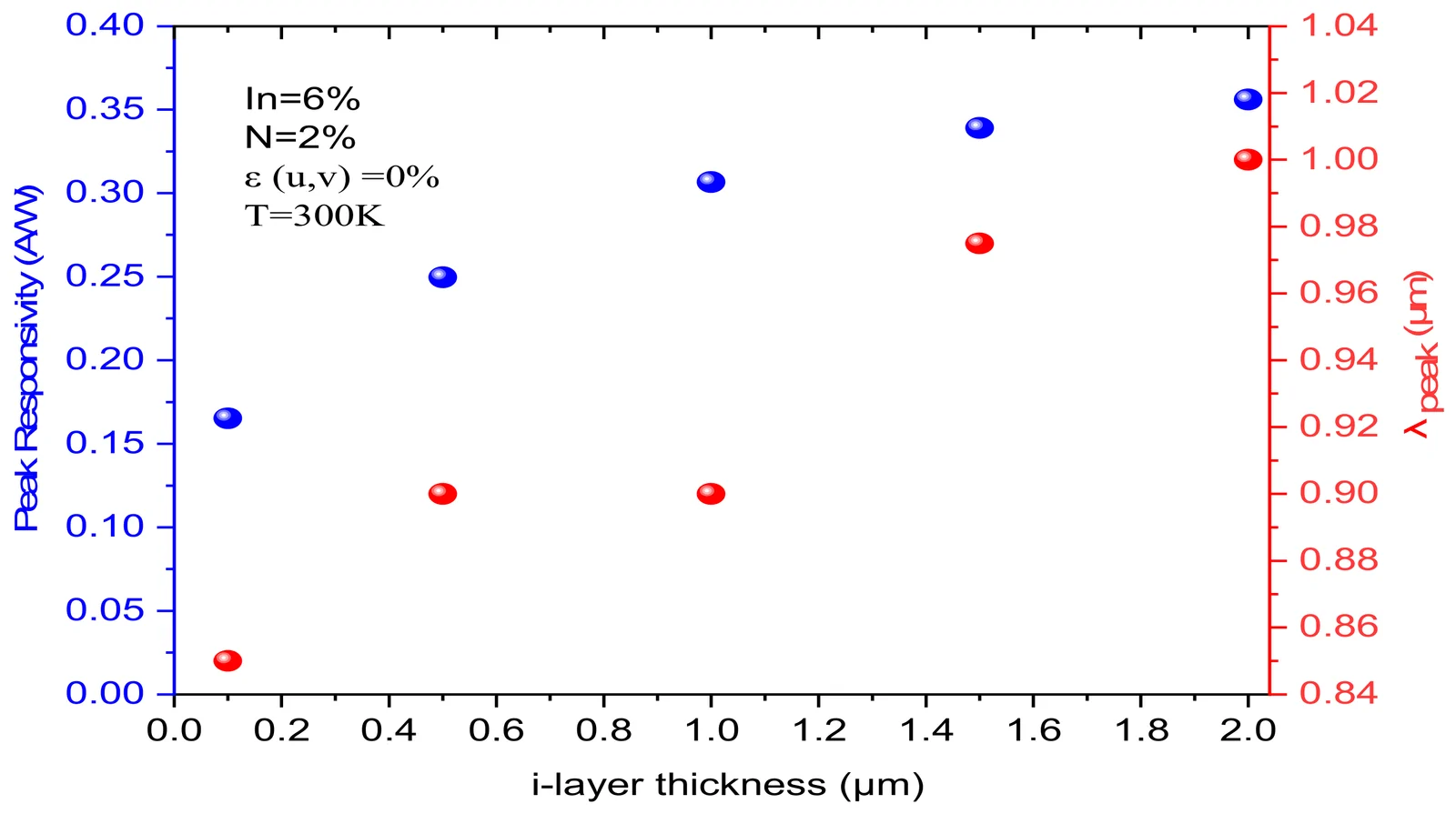
Peak responsivity and peak wavelength of the InGaAsN/GaAs p–i–n PD vary with different thicknesses of the absorbing i-layer (d_abs_).

Figure 6 illustrates that the peak R(λ) and λ_peak_ are influenced by d_abs_ in the InGaAsN/GaAs PD structure. The figure reveals that both the peak R(λ) and its related λ_peak_ grow as the d_abs_ increase from 0.1 to 2 µm. However, the peak R(λ) achieves a stable value for thicknesses over 1.5 μm. As the thickness of the absorbing layer increases, the probability of photon absorption rises accordingly.

However, beyond a thickness of 1.5 µm, the majority of incident photons are already absorbed, leading to a saturation in the device’s spectral response. Consequently, any further increase in thickness yields little to no additional improvement in performance.

When the d_abs_ exceeds the optimal limit, the recombination of carriers occurs, leading to an increase in material defects and a decline in the sensitivity of the photodiode. It is observed that when the absorber thickness exceeds 2 µm, the responsivity decreases rapidly. Therefore, it is crucial to find a compromise between the d_abs_ and recombination losses.

**Figure 7:**
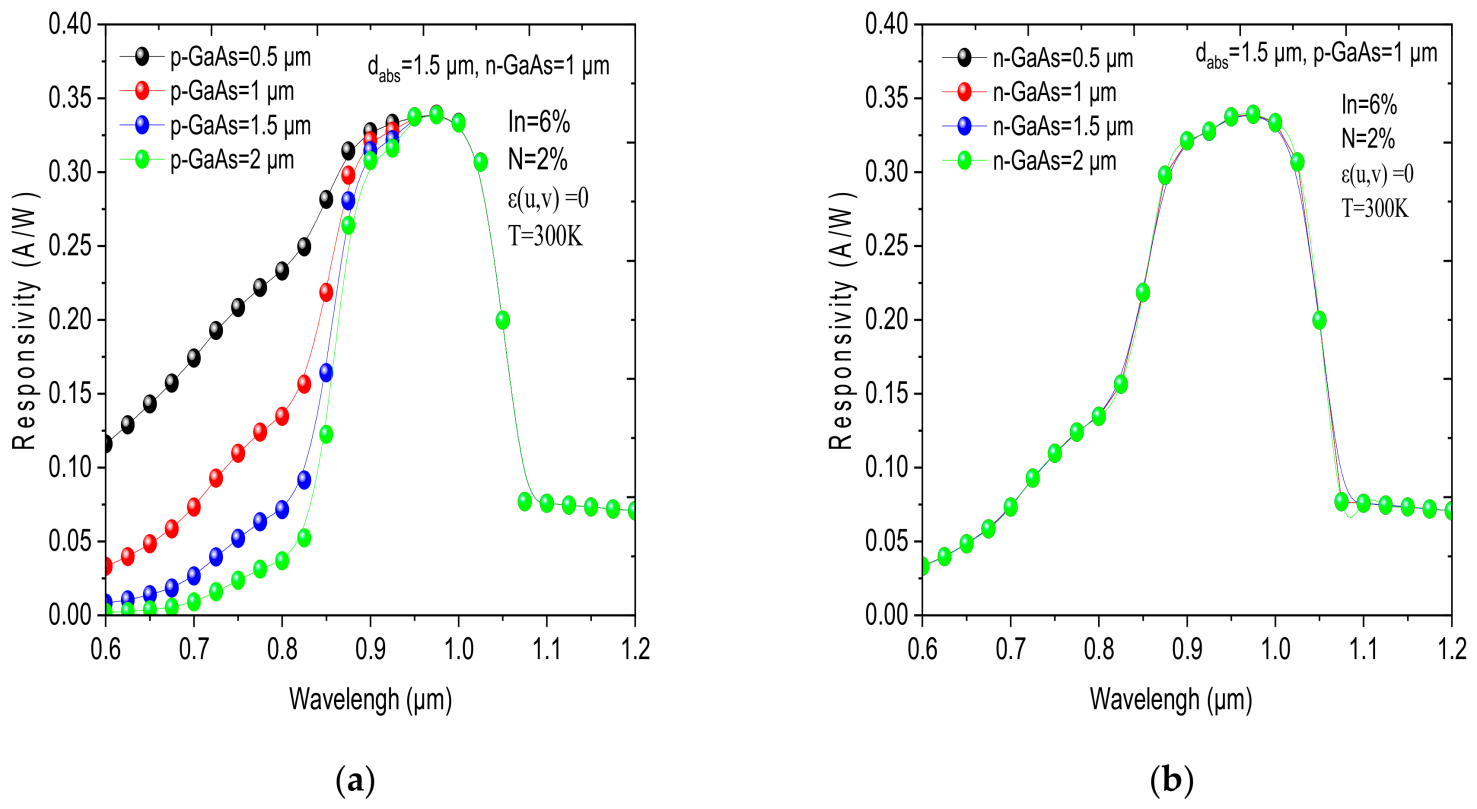
(**a**,**b**) Effect of responsivity as a function of wavelength for a p–i–n PD, (**a**) spectral responsivity with different p-GaAs thickness, (**b**) spectral responsivity for different n-GaAs thickness.

Figure 7a,b demonstrates the variation in R(λ) as a function of λ for different thicknesses w_p_ and w_n_ in a p-i-n photodetector. Figure 7a highlights the effect of thickness w_p_ on the sensitivity R(λ) of the InGaAsN/GaAs photodetector. The thickness w_p_ ranges from 0.5 to 2 µm, with d_abs_ = 1.5 µm. It is observed that the maximum of R(λ) remains constant even as w_p_ increases, while the response time of the sensitivity accelerates. This phenomenon can be attributed to the contribution of higher frequencies to the overall response. Furthermore, it is noted that increasing w_p_ leads to several effects, including a faster sensitivity response due to the displacement path of hole carriers, which results in a reduction in the spectral width of the sensitivity of the p-i-n photodiode, particularly for wavelengths below 0.9 µm. However, for wavelengths λ greater than 1 µm, w_p_ does not affect R(λ), as E_ph_ is lower than the bandgap energy of the GaAs material (E_ph_ < E_g,GaAs_). Consequently, the p-GaAs layer cannot absorb these photons, allowing them to pass through without interaction. This results in a diminished ability of the photodetector to convert incident photons into electrical current, significantly degrading its quantum efficiency in this wavelength range. The findings indicate that a higher w_p_ reduces photon absorption in the depletion layer, which lowers R(λ).

Figure 7b illustrates the effect of R(λ) from the InGaAsN/GaAs photodetector for various w_n_ values, while keeping dabs at 1.5 µm. The findings suggest that increasing w_n_ in the p-i-n PD design does not significantly affect sensitivity. In fact, the rise in w_n_ does not influence responsivity, as the generation and collection of carriers responsible for the photocurrent (J_ph_) primarily occur in the intrinsic region. This indicates that R(λ) is largely unaffected by w_n_, since most incident photons are absorbed in the intrinsic i-InGaAsN layer of the photodetector structure.

**Figure 8:**
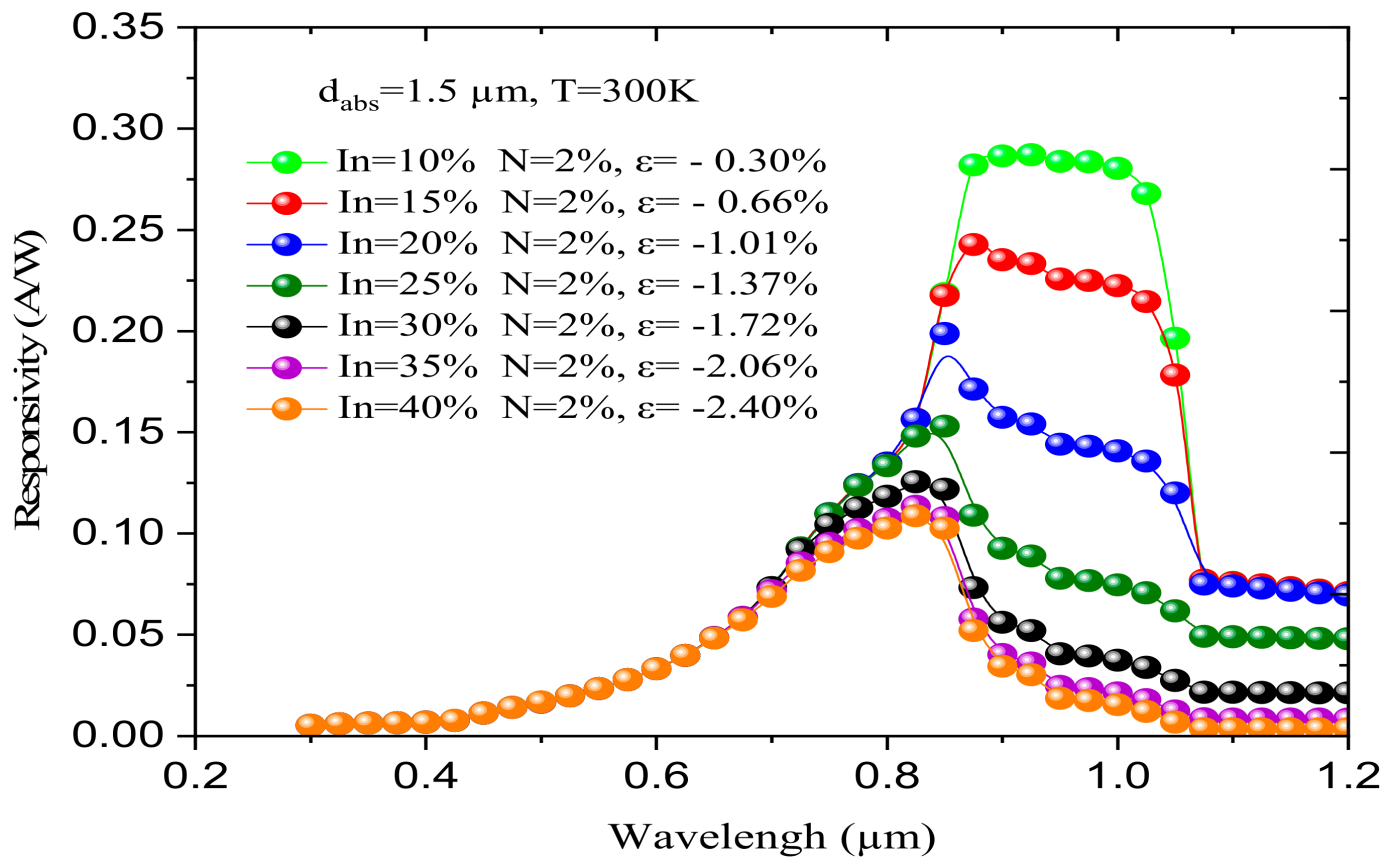
Responsivity of the InGaAsN/GaN p–i–n PD as a function of wavelength for different In concentrations.

The spectral characteristics of responsivity R(λ) in the p-i-n InGaAsN/GaAs PD structure at T = 300 K are depicted in Figure 8, showing In concentrations ranging from 10% to 40%, while the N concentration is held constant at 2% and dabs at 1.5 µm. As illustrated in Figure 8, with an In concentration of 10%, the peak of R(λ) reaches 0.287 A/W at λ = 0.925 µm. When the In concentration is increased to 20%, R(λ) decreases to 0.190 A/W at λ = 0.850 µm. Increasing the In concentration to 40% results in R(λ) dropping to 0.100 A/W at λ = 0.825 µm. These findings clearly indicate that a rise in In concentration significantly impacts the R(λ) of the p-i-n PD. This decline is attributed to increased mechanical stress, dislocation formation, and crystalline defects within the material. Such physical phenomena can lead to recombination between electrons and holes before their collection, resulting in reduced photocurrent, which diminishes responsivity and shifts the spectrum towards the infrared. This simulation aids in optimizing the indium concentration. It is observed that for an In concentration of 0.10, N = 0.02, and ε = 0.30%, the maximum responsivity exceeds 0.28.

**Figure 9:**
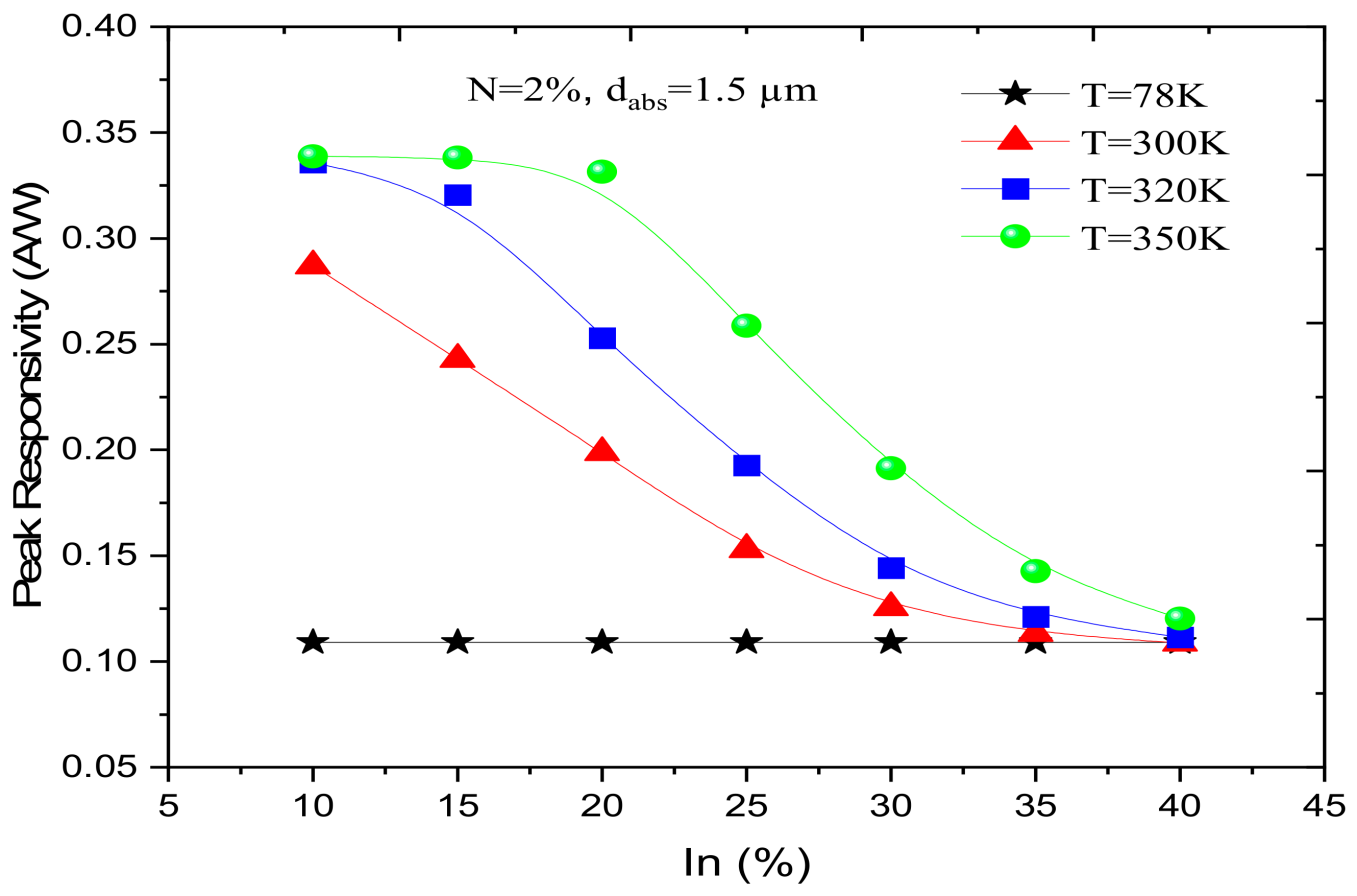
Variation in peak responsivity of a strained InGaAsN p-i-n PD with N = 2% as a function of temperature and In concentration.

Figure 9 illustrates how the peak R(λ) varies with the concentration of indium in the proposed InGaAsN p-i-n photodiode (N = 2% and d_abs_ = 1.5 µm) across different temperatures (T = 78 K, 300 K, 320 K and 350 K). At cryogenic temperature (T = 78 K), the responsivity remains constant at a lower plateau of approximately 0.109 A/W regardless of the variation in indium concentration, as the thermal energy is insufficient to activate many recombination mechanisms. Therefore, photogenerated carriers have a high likelihood of being collected, even with changes in indium concentration. In contrast, as the temperature increases from 300 K to 350 K, the peak responsivity increases significantly for low indium contents (In ≤ 25%), reaching a maximum of approximately 0.34 A/W at 350 K. This phenomenon can be explained by several physical factors: at 350 K, carriers exhibit considerable thermal energy, the absorption of incoming photons peaks, and the bandgap energy is around 1 eV. The bandgap narrowing is counterbalanced by the degradation of carrier transport at elevated indium contents. For higher indium fractions (In > 25%), the responsivity exhibits a pronounced decline, converging back to the 0.109 A/W plateau at In = 40%. This decrease is attributed to reduced carrier mobility and enhanced non-radiative Shockley–Read–Hall (SRH) recombination losses, which are typically exacerbated in highly compressively strained layers. Such transport limitations reduce the collection probability of photogenerated carriers before they reach the contacts, contributing to the observed drop in photocurrent J_ph_ and responsivity at elevated temperatures. Consequently, this numerical analysis provides valuable guidance for optimizing both indium concentration and operating temperature to maximize the optoelectronic performance of the photodiode.

**Figure 10:**
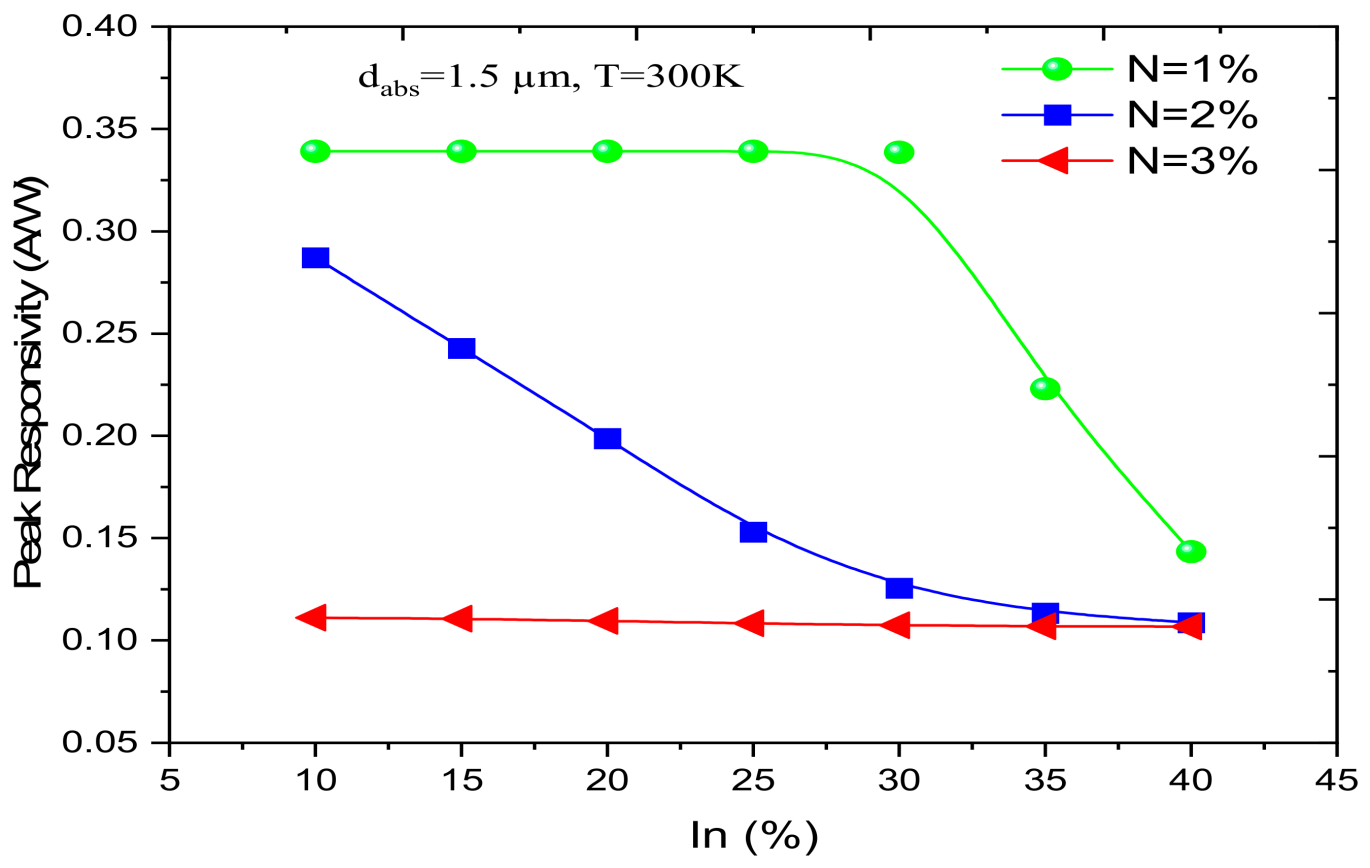
Variation in peak responsivity of a strained InGaAsN p-i-n PD at T = 300 K as a function of In Concentration for each N Concentration.

Figure 10 illustrates the evolution of the peak responsivity R(λ) under strain as a function of In and N concentrations for an absorber thickness of d_abs_ = 1.5 µm and T = 300 K. An increase in nitrogen (N) content leads to a pronounced decrease in the responsivity peak. Although nitrogen incorporation narrows the bandgap and extends the absorption threshold, it inherently degrades carrier transport properties due to localized states and strong scattering typical of dilute-nitride alloys. In the simulation framework, this degradation is reflected by reduced effective Shockley–Read–Hall (SRH) lifetimes and degraded carrier mobilities, which limit the collection efficiency of photogenerated carriers within the active layer. For N = 1%, the peak R(λ) remains stable at approximately 0.339 A/W for indium concentrations between 10 and 30%. However, beyond 30% indium concentration, the responsivity decreases progressively, dropping to 0.143 A/W at In = 40% due to substantial compressive strain. This degradation is associated with large compressive strain and the resulting lattice mismatch, which severely impairs effective carrier transport and reduces collection efficiency within the active layer.

For N = 2%, a continuous decrease is observed, starting from In = 10% and falling to 0.109 A/W at In = 40% due to degraded carrier collection under severe cumulative strain. At N = 3%, the peak R(λ) becomes virtually insensitive to variations in indium concentration. This saturation indicates that the severe reduction in effective carrier lifetime and mobility at elevated nitrogen fractions restricts the carrier diffusion length, thereby counteracting the optical bandgap tuning. Consequently, restricting the nitrogen content to N ≤ 2% under lattice-matched conditions represents the optimal design compromise to sustain efficient carrier collection and ensure high responsivity in the near-infrared regime.

**Figure 11:**
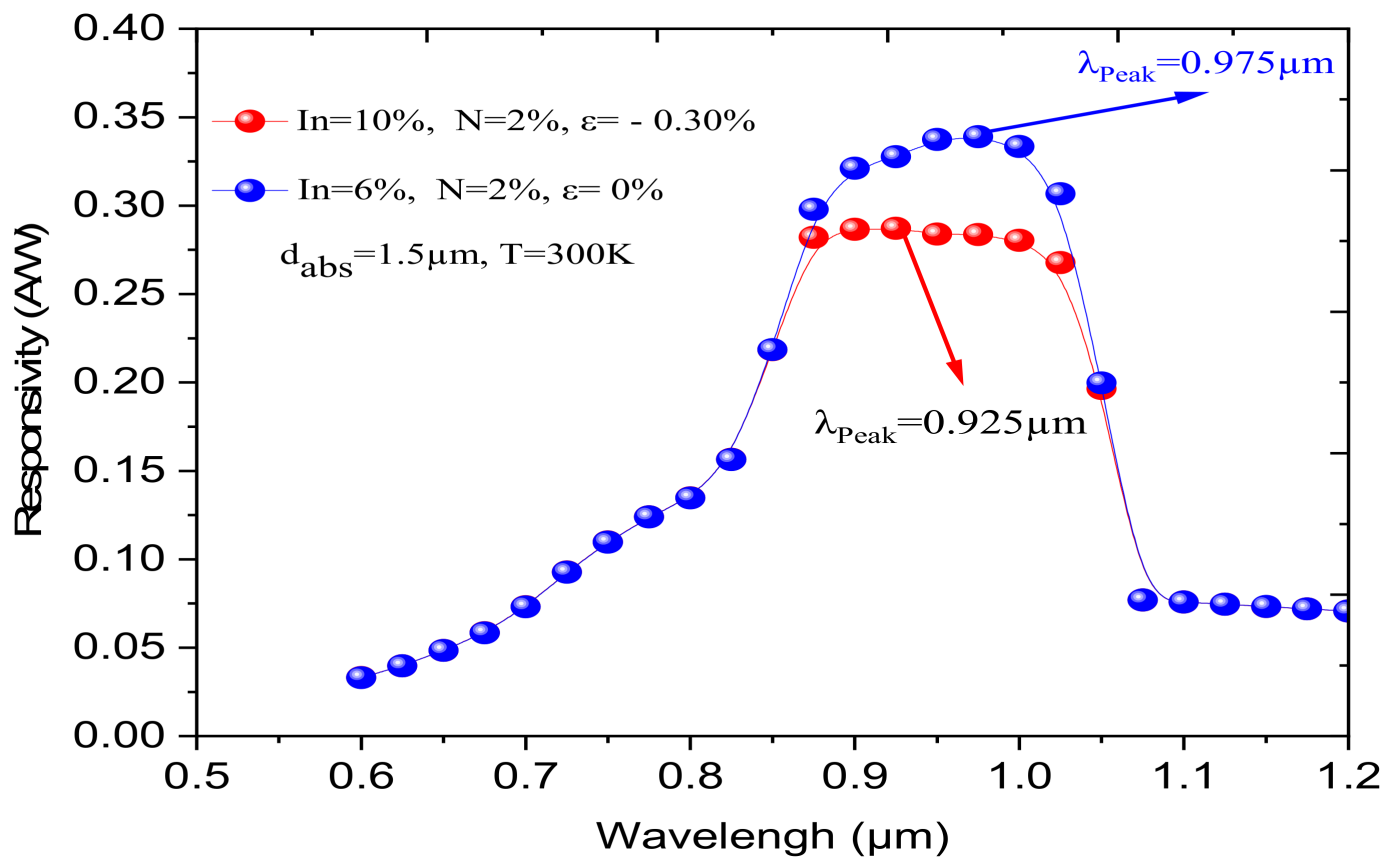
Characteristics of responsivity in unstrained and strained InGaAsN/GaAs p-i-n photodetector structures.

Figure 11 shows the spectral response R(λ) of the InGaAsN/GaAs PD structure at T = 300 K with d_abs_ = 1.5 µm. For concentrations of 6% In and 2% N, which correspond to a stable structure with ε(u,v) = 0%, the R(λ) achieves a value of 0.339 A/W at λ = 0.975 µm. When the InGaAsN/GaAs PD structure experiences strain due to an increase in In concentration to 10% (while keeping N at 2%), this rise in In concentration modifies the electronic characteristics of the structure, particularly by introducing strain into the crystal lattice. Consequently, the λ_peak_ of the PD’s responsivity shifts to shorter wavelengths, reaching 0.287 A/W as it moves from 0.975 to 0.925 µm, and the external quantum efficiency decreases to 38.47%.

In accordance with Planck’s relation (E_ph_ = hc/λ), the strain-induced bandgap (E_g_) narrowing lowers the fundamental optical absorption threshold, thereby enabling the absorption of lower-energy photons (longer wavelengths/red shift). However, excessive strain impairs overall device performance by modifying the band structure and degrading effective carrier mobility. Within the simulation framework, these transport limitations diminish carrier-collection efficiency, leading to a reduced responsivity R(λ) in the strained p-i-n PD compared to the unstrained (lattice-matched) p-i-n PD structure.

**Figure 12:**
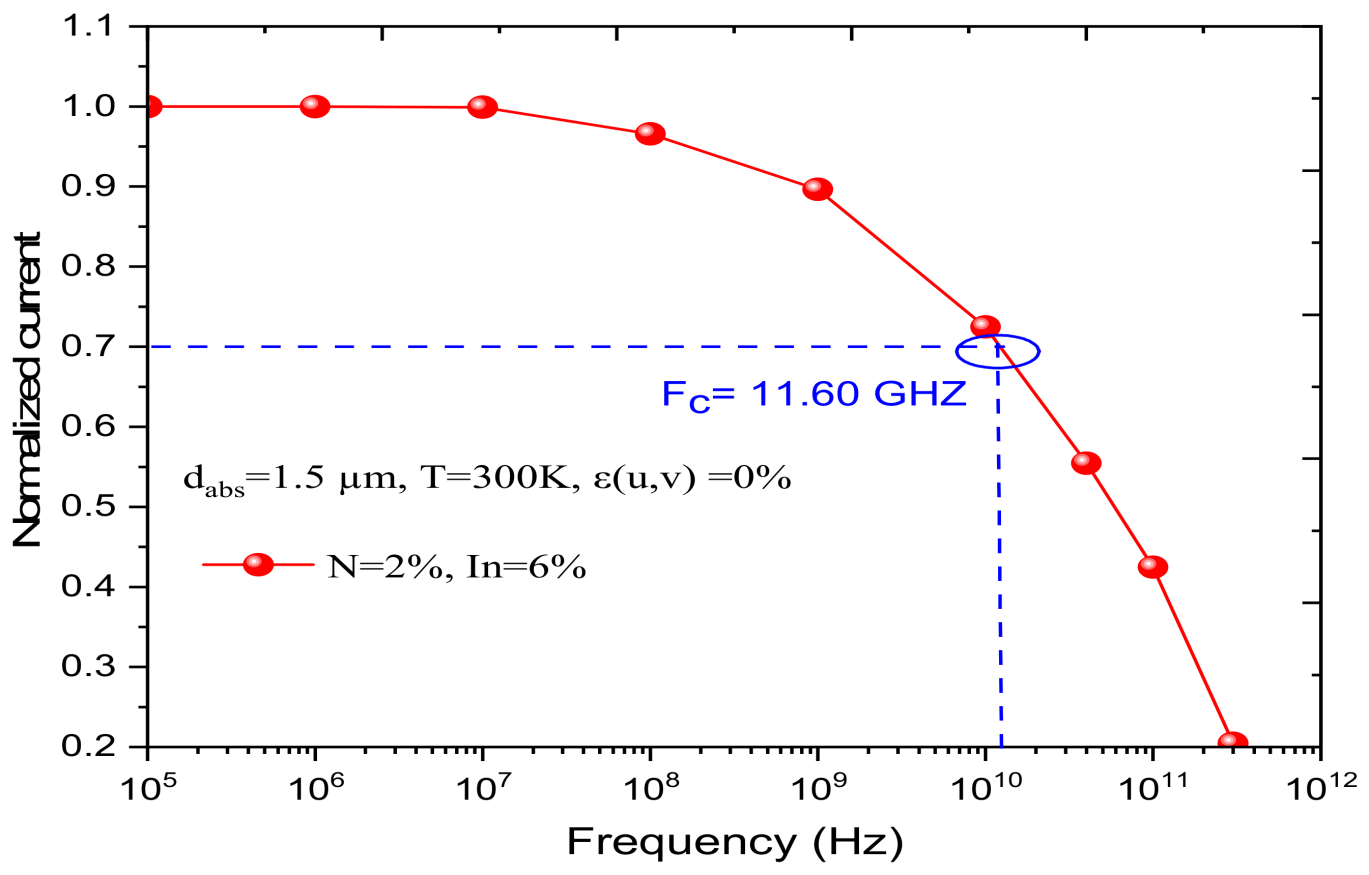
Normalized frequency response of the In_0.06_Ga_0.94_As_0.98_N_0.02_/GaAs p-i-n PD for N = 2% and In = 6%.

Figure 12 illustrates the frequency response of the unstrained InGaAsN/GaAs p-i-n PD (In = 6%, N = 2%), which exhibits a characteristic low-pass filter behavior. In this context, the normalized current, calculated relative to the maximum current, starts to decline when the frequency exceeds 10^7^ Hz and becomes negligibly small at 10^9^ Hz. At high frequencies, the charge carriers (electrons and holes) in the photodiode cannot traverse the active region, reducing carrier-collection efficiency and decreasing capacitive reactance, which in turn diminishes the measured current. The F_c_, where the response decreases by a factor of √2, is 11.60 GHz for an N concentration of 2% and an In concentration of 6%.

**Figure 13:**
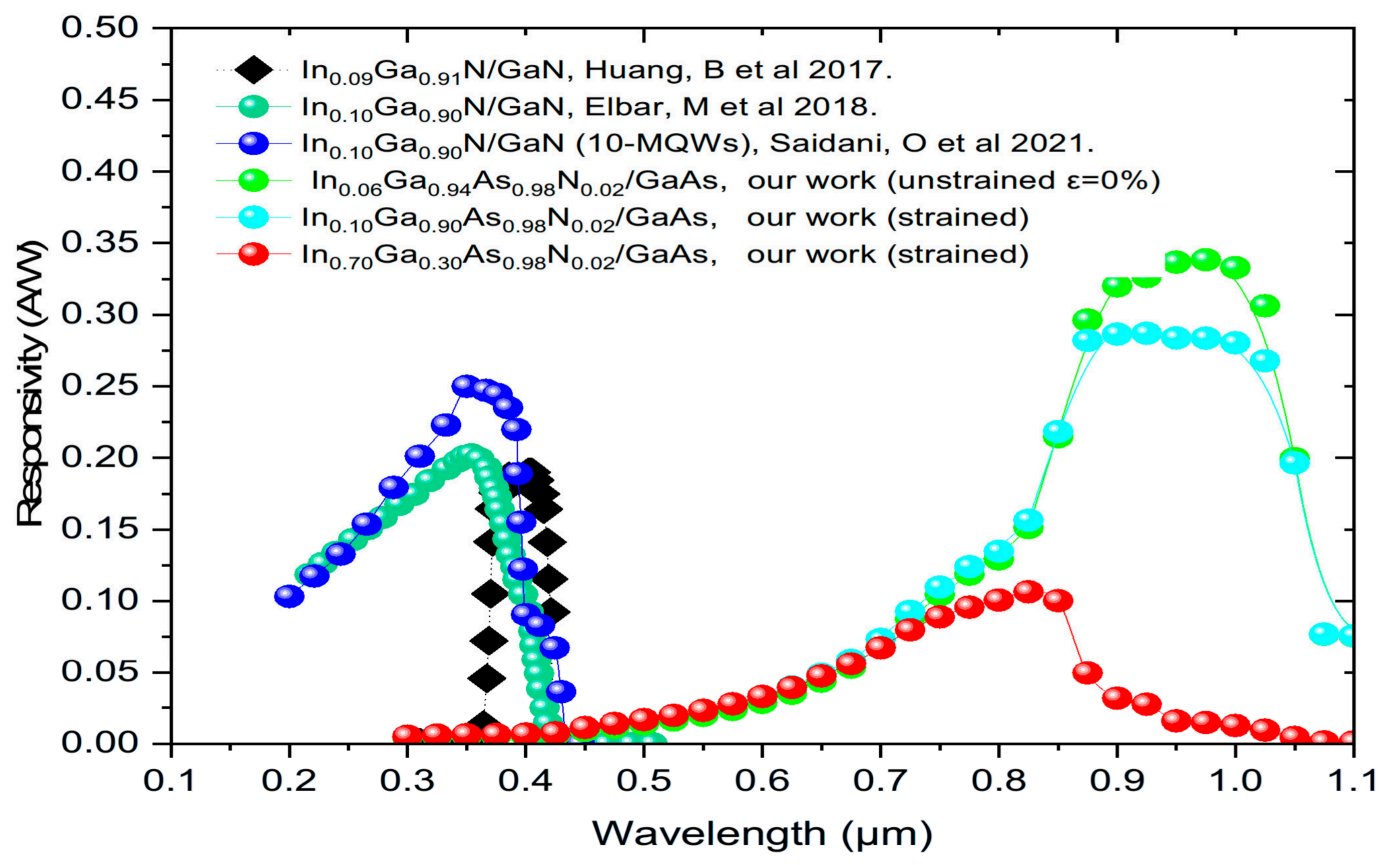
In order to evaluate the sensitivity of our results against the literature [16,18,36].

Figure 13 compares the spectral response (responsivity) of our structures with data from the literature [16,18,36]. The lattice-matched configuration (In = 6%, N = 2%, ε= 0% and T = 300 K, green curve) exhibits an optimal responsivity of 0.339 A/W near λ≈ 1 µm. For the strained structure (In = 10%, N = 2%), subjected to a compressive strain of ε = −0.30%, the responsivity reaches approximately 0.30 A/W at λ = 0.950 µm. By comparison, ternary InGaN-based photodetectors reported in references [16] (In = 9%) and [18] (In = 10%) show lower performance, with a peak value of 0.19 A/W at 0.400 µm and an external quantum efficiency exceeding 20% at 0.350 µm, respectively. Finally, reference [36] incorporates In_0.10_Ga_0.90_N/GaN quantum wells within the absorption region.

This configuration achieves a peak responsivity of 0.25 A/W at an operating wavelength of 0.350 µm. Compared to the structure reported in ref. [36], our optimal In_0.06_Ga_0.94_As_0.98_N_0.02_ design achieves a relative improvement of 26.25% while operating in the near-infrared (NIR) regime near λ ≈ 1 µm. Consequently, the incorporation of dilute- nitride quaternary alloys enables the design of high-responsivity, robust, and reliable photodiodes in this spectral window. Finally, our third structure (In = 70%, N = 2%) yields a peak responsivity below 0.10 A/W at λ = 0.840 µm, which is constrained by the substantial compressive strain present in the active layer. Table 2 summarizes the performance of our proposed photodetector in comparison with benchmark simulations and data from the literature [14,16,17,18,36,37]. Under a 2 V reverse bias and with an absorption layer thickness of d_abs_ = 1.5 μm, our lattice-matched design (In = 6%, N = 2%, ε = 0%, T = 300 K) achieves a maximum responsivity of 0.339 A/W at λ = 0.975 µm and a cutoff frequency F_c_ of 11.60 GHz.

The inclusion of wide-bandgap III-nitride counterparts (such as InGaN/GaN) [14,16,17,18,36] is intended not to claim a direct enhancement in responsivity metrics across disparate regimes, but rather to contextualize the device within the broader evolution of III–V nitride technology. Historically centered on the ultraviolet/blue spectral domain (0.343–0.402 µm) with peak responsivities ranging from 0.190 to 0.250 A/W and bandwidths up to 4 GHz.

In contrast, in the near-infrared regime, our device significantly outperforms the dilute-nitride photodiode reported in ref. [37], which exhibits a responsivity of 0.032 A/W at 1.00 µm. Our proposed device provides a more than tenfold enhancement in responsivity, together with a high cutoff frequency of 11.60 GHz. This improved performance is attributed to the wide-bandgap GaAs contact and cladding layers, which act as transparent window layers that minimize parasitic surface absorption and maximize light delivery into the InGaAsN active region. Concurrently, the optimized balance between carrier transit time and reduced junction capacitance supports high- frequency operation while preserving quantum efficiency, highlighting the potential of such dilute-nitride architectures for high-speed, high-efficiency photodetection.

## 4. Conclusions

This research details the numerical simulation and modeling of an In_0.06_Ga_0.9_4As_0.98_N_0.02_/GaAs p-i-n photodiode using the Atlas-Silvaco simulator. We systematically investigated the influence of layer thickness variations (absorption layer, n-GaAs, and p-GaAs) on device performance. For the lattice-matched configuration (In = 6%, N = 2%, ε = 0%), a peak responsivity R(λ) of 0.339 A/W was achieved at λ_peak_ 0.975 µm, corresponding to an external quantum efficiency (QE) of 43%.%. Furthermore, a cutoff frequency F_c_ of 11.60 GHz was obtained under a 2 V reverse bias with an optimal absorber thickness of d_abs_ = 1.5 μm. The effect of increasing the indium concentration up to 10% at a fixed nitrogen concentration of 2% was also examined, inducing a compressive strain of ε = −0.30%. A comparative analysis shows that the lattice-matched design provides improved responsivity, confirming the benefit of minimizing strain-induced degradation to enhance steady-state optoelectronic performance. Operating near 1 µm, this optimized design demonstrates remarkable potential for high-speed optoelectronic applications, including optical fiber communications, LiDAR systems, and high-precision infrared sensing. Finally, epitaxial growth and experimental characterization (namely I-V measurements and spectral response testing) constitute the intended outlook and future work of this study.

## Figures and Tables

**Table 2:** Comparison of the results of our study with previously reported p-i-n PD.

**Structure Photodiode p-i-n**	**Peak Responsivity** (A/W)	Wavelength (µm)	Cutoff Frequency	Ref.
In0.11Ga0.89N/GaN	0.206	0.380	not reported	[14]
In0.09Ga0.91N/GaN	0.190	0.402	not reported	[16]
In0.10Ga0.90N/GaN MQW	not reported	not reported	300 MHz	[17]
In0.10Ga0.90N/GaN	0.200	0.343	400 MHz (dabs = 0.1 µm)	[18]
In0.10Ga0.90N/GaN	0.244	0.384	4 GHz (dabs = 1.5 µm)	[18]
In0.10Ga0.90N/GaN (10-MQW)	0.25	0.35	460 MHz	[36]
Ga0.90In0.10N0.033As0.967/GaAs	0.032	1.000	not reported	[37]
In0.06Ga0.94As0.98N0.02/GaAs	0.339	0.975	11.60 GHz	our work

## Data Availability

The original contributions presented in the study are included in the article; further inquiries can be directed to the corresponding author.
